# Genome-Wide Identification and Analysis of *MYB* Transcription Factor Family in *Hibiscus hamabo*

**DOI:** 10.3390/plants12071429

**Published:** 2023-03-23

**Authors:** Dina Liu, Chunsun Gu, Zekai Fu, Zhiquan Wang

**Affiliations:** 1 Institute of Botany, Jiangsu Province and Chinese Academy of Sciences, Nanjing 210014, China; 2 College of Forest Sciences, Nanjing Forestry University, Nanjing 210037, China; 3 Jiangsu Key Laboratory for the Research and Utilization of Plant Resources, Nanjing 210014, China

**Keywords:** MYB family, transcription factors, abiotic stress, semi-mangrove plant, *Hibiscus hamabo*

## Abstract

MYB transcription factors constitute one of the largest gene families in plants and play essential roles in the regulation of plant growth, responses to stress, and a wide variety of physiological and biochemical processes. In this study, 204 MYB proteins (HhMYBs) were identified in the *Hibiscus hamabo* Sieb. et Zucc (*H. hamabo*) genome and systematically analyzed based on their genomic sequence and transcriptomic data. The candidate HhMYB proteins and MYBs of *Arabidopsis thaliana* were divided into 28 subfamilies based on the analysis of their phylogenetic relationships and their motif patterns. Expression analysis using RNA-seq and quantitative real-time PCR (qRT-PCR) indicated that most *HhMYB*s are differentially regulated under drought and salt stresses. qRT-PCR analysis of seven selected *HhMYB*s suggested that the HhMYB family may have regulatory roles in the responses to stress and hormones. This study provides a framework for a more comprehensive analysis of the role of MYBs in the response to abiotic stress in *H. hamabo*.

## 1. Introduction

*Hibiscus hamabo* Sieb.et Zucc. (*H. hamabo*) is a deciduous shrub native to the coastlines of Korea, Japan, and China that is currently considered an endangered plant in the Zhejiang Province of China [1,2,3]. As a semi-mangrove species, it is adaptable to pronounced changes in ecological characteristics in land–sea transitions [2,4], with a strong adaptability to saline alkali soil [5]. Therefore, *H. hamabo* plays an important role in the improvement of saline–alkali lands and has been widely applied in the afforestation of coastal beaches in environmental governance efforts in recent years [5]. *H. hamabo* is an excellent material for the study of plant salt tolerance. At present, studies on salt tolerance in *H. hamabo* have mainly been based on morphological observations: investigations of the physiological and molecular mechanisms involved [6,7,8,9]. Previously, we presented the genome sequencing of *H. hamabo* using PacBio, Illumina, and Hi-C sequencing technology and analyzed its salt tolerance mechanism based on genome and transcriptome data [2].

The MYB transcription factor family is one of the largest in plants and plays key roles in the regulation of plant growth and stress [10,11]. MYB family members have highly conserved domains forming three α-helices in three-dimensional space [10]. The second and third helices form a “helix-turn-helix” structure with a hydrophobic core containing three tryptophan residues, which is the DNA binding site [10]. According to the number of adjacent imperfect repeats, MYB genes can be divided into four gene subfamilies, namely 1R-MYB, 2R-MYB, 3R-MYB, and 4R-MYB [12].

MYB transcription factors have been reported to alleviate or eliminate plant stress-related damage through their signal transduction network, which regulates targeted genes to initiate physiological and biochemical responses [13,14]. Abscisic acid (ABA) and salt can induce transcription levels of *AmMYB1*, and the overexpression of *AmMYB1* in tobacco improves the tolerance to salt stress [15]. Overexpression of the wheat R2R3-MYB gene members *TaMYB32*, *TaMYB33*, *TaMYB56-B*, and *TaMYB73* in *Arabidopsis thaliana* (*A. thaliana*) significantly improved salt tolerance [11]. All the genome data of *A. thaliana* provided the possibility for the description and classification of the MYB gene family in plants [16]. Since then, scientists have identified the MYB family from different species and clarified their biological function and that of related genes by molecular biological and other means [12,17,18]. In this study, all MYB genes detected in the genome of *H. hamabo* and their chromosomal distribution, phylogenetic relationships, gene structural organization, and protein motif content were analyzed. Furthermore, their expression in response to stress stimuli was analyzed using previously published transcriptomic data of *H. hamabo*, which was validated for selected MYB members by comparative RT-qPCR. This work provides a preliminary framework for the identification of candidate MYB genes mediating stress responses, especially to salt stress in *H. hamabo*. The candidate genes identified could be useful both in future studies of the underlying mechanisms of salt tolerance in *H. hamabo* and as potential markers in subsequent *H. hamabo* breeding.

## 2. Materials and Methods

### 2.1. Identification of MYB Gene Members in the H. hamabo Genome

HMMER software (v. 3.1) was used with the hidden Markov model (HMM) of the MYB domain (PF00249), obtained from the Pfam database (http://pfam.xfam.org/, accessed on 18 March 2021), to identify MYB genes in the *H. hamabo* genome (Bioproject accession number: PRJNA759075) with E-values < 0.001 [2,4]. ClustalX software (v2.0) was used for alignment of protein sequences and eliminating redundant sequences. The Pfam website (http://pfam.xfam.org/search#tabview=tab1, accessed on 18 March 2021) was used to check the MYB domain structure, and sequences with a typical conserved domain were identified as *H. hamabo* MYB members (*HhMYB*), which were used in further analyses.

### 2.2. Phylogenetic, Intron-Exon Structure, and Motif Compositional Analyses of HhMYBs

The phylogenetic tree of MYB proteins of *H. hamabo* and *A. thaliana* was constructed using MEGA 7.0 [19] with the nearest neighbor (NJ) method and 1000 bootstraps. The gene structure was visualized using Gene Structure Display Server (GSDS) [20]. MEME (http://meme.nbcr.net/meme, accessed on 18 March 2021) was used for the analysis of conserved motifs with the maximum number of motifs set at 10.

### 2.3. Chromosome Mapping of HhMYBs

The mapping of *HhMYB*s to chromosomes was based on their annotation information from the H. hamabo genome and was visualized with CIRCOS [21].

### 2.4. Expression Pattern Analysis of HhMYBs Obtained from Transcriptome Sequencing

The expression profile data for expression analysis of *H. hamabo* in different periods of salt and drought treatment were obtained from RNA-Seq data [22]. Fragments per kb per million reads (FPKM) were used as the standard for expression pattern, and the omicshare tool was used to generate the associated heatmap figures (https://www.omicshare.com/tools/Home/Soft/heatmap, accessed on 18 March 2021).

### 2.5. Plant Materials and Stress Treatment

The plant materials used in this study were collected from Institute of Botany, Jiangsu Province, and the Chinese Academy of Sciences (Nanjing Botanical Garden Mem. Sun Yat-Sen). Healthy and well-developed *H. hamabo* seeds were placed in a refrigerator at 4 °C for vernalization for 20 days. The seeds were then treated with concentrated sulfuric acid for 15 min and then rinsed thoroughly with running water before planting in plastic pots with a substrate composed of peat and vermiculite (1:1) and placing in a phytotron with a photoperiod of 16/8 h and day/night temperatures of 24/20 °C. Seedlings with 8–10 true leaves were transplanted into 1/2 MS solution for 7 days, followed with abiotic stress and hormone treatments.

High and low temperature stress treatments consisted of exposing the seedlings to 42 °C or 4 °C, respectively. A certain number of seedlings were exposed in 400 mM NaCl or 500 mM mannitol for high salt or drought treatment, respectively. Other seedlings were sprayed with 200 μM salicylic acid (SA), 1 mM methyl jasmonate (MeJA), or 50 μM abscisic acid (ABA) until the leaves were completely moist. The youngest fully expanded leaf from each plant was collected at 1, 2, 6, 12, and 24 h after treatment and then frozen in liquid nitrogen before storage at −80 °C. The youngest fully expanded leaf from each plant of untreated seedlings was similarly collected as control material. Three biological replicates with nine different plants, three plants in a group, were produced for each treatment.

### 2.6. Expression Analysis of the HhMYB Genes by qRT-PCR

Total RNA was extracted with a total RNA extraction kit (Shanghai PuDi Biotech Co., Ltd., Shanghai, China), and cDNA was synthesized with HiScript^®^ III 1st Strand cDNA Synthesis kit (Vazyme, Nanjing, China), both procedures following their respective manufacturer recommendations. The Genscript tool (https://www.genscript.com/tools/pcr-primers-designer, accessed on 20 March 2021) was used for designing primers for qRT-PCR (Table S1). qRT-PCR was performed using a StepOnePlus TM Real-Time PCR system (Applied Biosystems, Foster City, CA, USA). The reaction mixture contained 2 µL diluted cDNA, 10 µL 2 × SYBR Green Master Mix (Bimake, Houston, TX, USA), 0.4 µL ROX (Dye I), 1 µL each of forward and reverse primer, and ddH_2_O to a total volume of 20 µL. The PCR procedure utilized an initial denaturation step set as 95 °C for 10 min, followed by 40 cycles of 95 °C for 15 s, 60 °C for 30 s, and 72 °C for 30 s. Melting curve analyses of the amplified products were conducted at 60–95 °C. The *Actin* was selected as a reference gene: forward primer: 5′-GGCACCTCTCAACCCCAAGG-3′, reverse primer: 3′-GAGAGAACGGCCTGGATGGC-5′ [5]. Three technical replicates were prepared for each extract, and the quantitative results were analyzed using the 2^−ΔΔCT^ method.

## 3. Results

### 3.1. Identification of MYB Gene Family Members in H. hamabo

In order to identify MYB family genes in the genome of *H. hamabo*, we used the hidden Markov model profile (HMM) of the MYB domain (PF00249) to conduct an HMM-search based against the predicted gene coding sequences. A total of 204 genes were obtained with E-values < 0.001 and labeled as *HhMYB1*-*HhMYB204* (Table S2). The length range of the HhMYB sequences was from 300 aa to 1731 aa, of which HhMYB186 had the longest amino acid sequence. In addition, the molecular weights of HhMYBs ranged from 32.81 kDa (HhMYB4) to 196.98 kDa (HhMYB186), and the range of theoretical isoelectric points was 4.51 (HhMYB195) to 10.01 (HhMYB30) (Table S2).

### 3.2. Phylogenetic Analysis of HhMYBs

**Figure 1:**
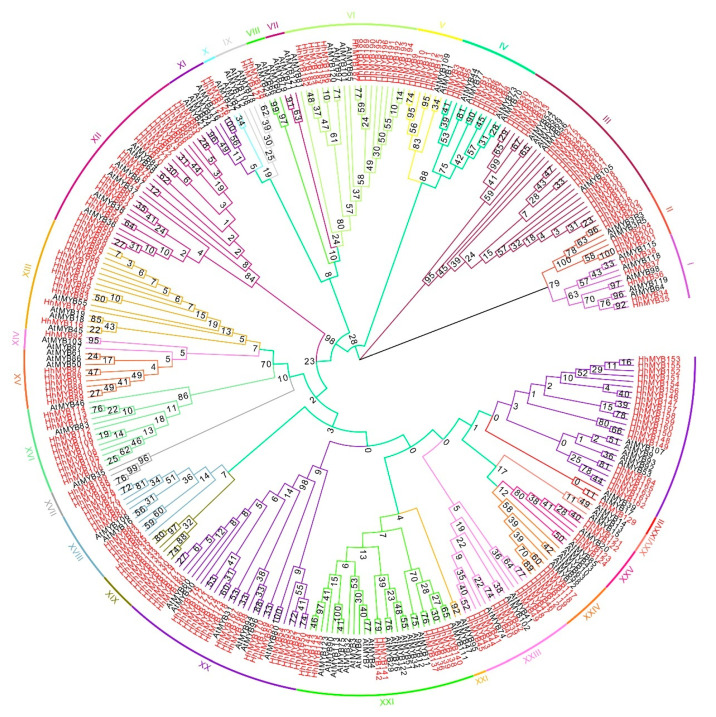
Phylogenetic tree of MYB family members of *H. hamabo* and *A. thaliana* using the NJ method. The prefix At was used before the names of the *Arabidopsis* MYBs. The subfamilies were represented by different colors.

In order to analyze the phylogenetic relationships of HhMYBs, a phylogenetic tree was constructed from their predicted coding sequences. MYBs of *A. thaliana* (*AtMYB*s) were included for comparison (Figure 1). The candidate HhMYBs and AtMYBs were clustered within 28 subfamilies but showed differing clustering patterns among the subfamilies, suggesting the divergence of the MYB family during the evolution of these species. The XX subfamily had the most HhMYBs (22), whereas the XIV and XXVII subfamilies each contained only one member, and VII, VIII, X, XXII, and XXVI subfamilies contained no HhMYBs. It is worth noting that several MYB subfamilies were found to contain a greater number of HhMYBs relative to AtMYBs. For example, the III subfamily contains 20 HhMYBs but only 5 AtMYBs. Conversely, some subfamilies consisted exclusively of AtMYBs, indicating that these genes may be lost during the evolution of *H. hamabo*.

### 3.3. Conserved Motif and Gene Structure Analysis

In order to determine if the hierarchical clustering in the phylogenetic tree reflected differences in conserved motifs and/or gene structure, the candidate HhMYBs and AtMYBs were analyzed for motif content and their genes for intron/exon organization. The results (Figure S1) showed that motif 1 was highly conserved and present in most MYB members in *H. hamabo* and *Arabidopsis*, while other motifs showed a larger variability. Notably, the profiles of the HhMYB motif contents differed between the branches of the evolutionary tree and were relatively consistent within each branch. Only a few HhMYB proteins located in the same branch contained different motif profiles. Structural analysis revealed that the majority of HhMYB members (147) had a typical structure containing two introns (Figure S1). However, HhMYBs 1, 6–8, and 10–13 had no introns, whereas other members contained 1 to 15 introns, and HhMYBs with the same number of introns were grouped into a similar subclade.

### 3.4. Chromosome Mapping Analysis of MYBs in H. hamabo

The 204 *HhMYB* genes were mapped onto the 46 chromosomes using their annotated information provided from the earlier genomic analysis of *H. hamabo*. This showed that *HhMYB*s were unevenly distributed across the 46 chromosomes in a manner independent of chromosomal length (Figure S2). The largest number of *HhMYB*s were located on chromosome 3, with a total of 11 *HhMYB* genes. Some chromosomes contained only one *HhMYB*, such as chromosomes 27, 33, 38, 43, and 45.

### 3.5. Analysis of HhMYB Gene Expression Patterns under Salt and Drought Stress

**Figure 2:**
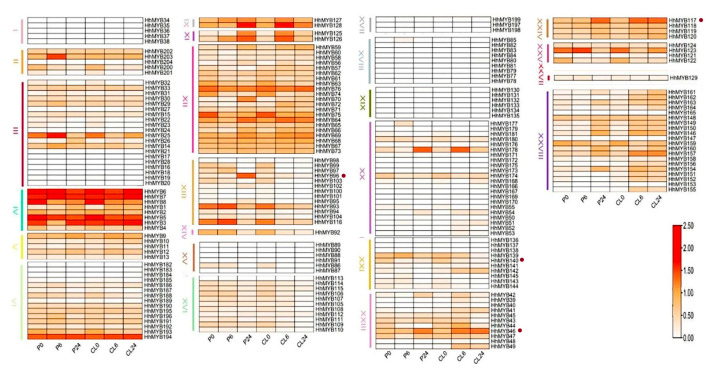
Expression pattern of *MYB* genes in *H. hamabo* under salt and drought stress as determined from RNA-Seq data. The expression level was represented by color: red, higher expression; white, lower expression. HhMYBs with a dot were specifically mentioned in the manuscript. P0, P6, P24, CL0, CL6, and CL24 represent treatments for 0 h, 6 h, and 24 h under PEG treatment and 0 h, 6 h, and 24 h under NaCl treatment, respectively.

In order to gain insight into the potential function of *HhMYB*s in response to saline and drought stress, we analyzed the expression patterns of *HhMYB*s using transcriptomic data obtained from *H. hamabo* subjected to these conditions (Figure 2) [22]. *HhMYB* genes in subfamilies XIX, XVIII, XVII, and I were not detected in any sample, whereas other subfamilies showed significant differences in expression patterns in response to the stress treatments. Several *HhMYB*s showed similar response trends to the two stresses. For example, the expression levels of *HhMYB46* of XXIII and *HhMYB117* of XXIV were up-regulated under both stresses, while the expression levels of *HhMYB140* of the XXI subgroup was down-regulated under both stresses, suggesting that these genes may play roles in both drought and salt stress. However, some genes, such as *HhMYB96* of the subgroup XIII, displayed different expression patterns under the two stresses. The expression level of *HhMYB96* increased under drought stress but decreased under salt stress, suggesting the differential deployment of this gene in response to temperature and drought stresses in *H. hamabo*. In addition, *HhMYB* genes in the same evolutionary branch showed different expression characteristics, indicating their functional differentiation.

### 3.6. qRT-PCR Analysis of Expression Patterns of HhMYBs under Temperature and Hormonal Treatments

**Figure 3:**
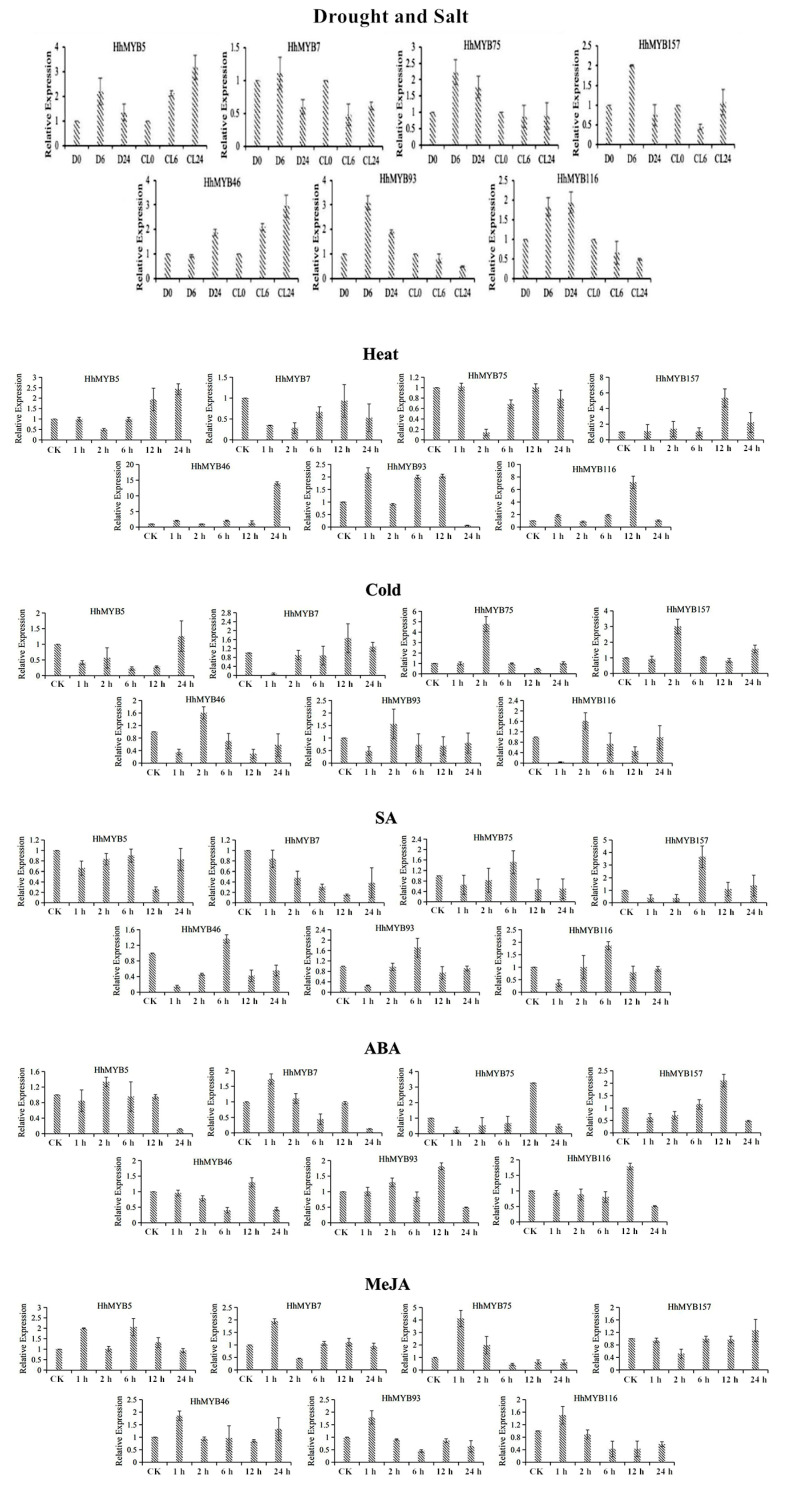
Expression pattern of *HhMYB* genes in *H. hamabo* under 500 mM mannitol (Drought), 400 mM NaCl (Salt), 42 °C (Heat), 4 °C (Cold), 200 μM salicylic acid (SA), 50 μM abscisic acid (ABA), or 1 mM methyl jasmonate (MeJA) treatments. D0, D6, D24, CL0, CL6, and CL24 represent treatments for 0 h, 6 h, and 24 h under drought stress and 0 h, 6 h, and 24 h under salt stress, respectively.

Seven selected *HhMYB* genes with relatively large fold changes under the PEG and NaCl treatments were tested for their response to salt and drought stress; high and cold temperatures; and SA, ABA, and MeJA treatments by qRT-PCR analysis. It can be seen from Figure 3 that the expression of these genes underwent significant changes under the different stress treatments. The expression trends of HhMYB75 were essentially opposite under low and high temperature conditions. All the selected *HhMYB*s were responsive to the various hormone treatments but showed differing expression trends, indicating that *HhMYB* genes are differentially deployed in response hormones.

## 4. Discussion

MYBs constitute one of the largest transcription factor families in plants, with a conserved MYB–DNA binding domain composed of about 52 amino acids at the N-terminus [23]. MYB transcription factors play a key role in plant development, secondary metabolism, hormone signal transduction, disease resistance, and abiotic stress tolerance [11,24,25]. With the increasing number of sequenced plant genomes, members of the MYB family have been systematically identified in various plant species [17,26]. For example, 197 candidate MYBs were identified in *Arabidopsis*, and a different number of candidate MYBs has also been identified in other species, such as 155 in rice and 245 in *Helianthus annuus* L. [27,28]. However, the MYB gene family in *H. hamabo* has not been reported, and its functions remain unclear. *H. hamabo*, with strong adaptability to saline alkali soil and seawater immersion, can adapt to the land–sea transition zone. Because *H. hamabo* is a good material for studying plant resistance, MYB is an important plant regulator. Genome-wide identification and analysis of MYB transcription factor family in *H. hamabo* were performed for the first time in this study. The results of this study showed that *H. hamabo* has a family of 204 *MYB*s, which may be due to large-scale fragment replication in the genome of *H. hamabo* [2].

Based on a phylogenetic analysis, the 204 candidate HhMYB proteins and AtMYBs were divided into 28 subfamilies. Almost all of these subfamilies contained both HhMYBs and AtMYBs, but in differing proportions. HhMYBs clustered in the same subfamily, in many cases, shared similar and highly conserved profiles of MYB motifs, suggesting that they may be functionally related. Only a few HhMYB proteins located in the same branch contained different motif profiles, indicating that these HhMYB members may have arisen as the result of functional differentiation during *H. hamabo* evolution. Additionally, HhMYB members have introns varying from 0 to 15, which are similar to those found in studies on, for example, *Hedychium coronarium* [29]. The results again indicated complex differentiation during evolution.

As a semi-mangrove plant, *H. hamabo* has a high resistance to saline and alkaline soils and drought and barren environments [8]. Some *Arabidopsis* MYB transcription factors have been reported to be involved in plant resistance to salt and drought stresses [30,31]. *AtMYB60* can influence the drought response by regulating the ABA signal transduction pathway [32]. The wheat *MYB33* was reported to enhance the salt and drought tolerance of transgenic *Arabidopsis* through its restoration of osmotic balance and increase in ROS-scavenging capabilities [33]. Here, the analysis of transcriptomic data indicated that several *HhMYB*s were involved in the plant responses to both saline and drought stresses. The expression patterns of the genes were different under drought or saline stress, suggesting that these *HhMYB*s may have regulatory functions in the tolerance to saline and drought stress and deserve further investigation.

Other biotic stresses, such as high and low temperatures, also seriously affect the growth of *H. hamabo*. MYBs have been reported to regulate the response and resistance to temperature stress. Transgenic rice overexpressing *OsMYB3R-2* exhibited an enhanced cold tolerance as well as an increased cell mitotic index [34]. Therefore, the expression levels of *HhMYB*s under temperature stress were also analyzed in this study. The expression trends of *HhMYB75* were essentially opposite under low and high temperature conditions. Similarly, AtMYB104 has been reported to be down-regulated by cold stress but up-regulated under high temperature, while the expression trend of *AtMYB81* displayed the opposite [12].

The involvement of MYBs in plant responses to environmental factors may be mediated by hormones [35]. *AtMYB*s *2*, *13*, *15*, and *101* have been shown to respond to ABA, and the ABA regulation of *AtMYB2* can influence the response to saline stress [36]. Many potato *StMYB* genes were induced under ABA, GA, and IAA treatments [36]. The results in this study showed that a high proportion of *HhMYB*s are responsive to ABA, SA, or MeJa, but with differing levels of intensity and at different time points after hormonal stimulation. This study provides a reference for future research into the role of MYB transcription factors in the response of *H. hamabo* to abiotic stresses.

## 5. Conclusions

Based on the importance of the MYB family in plants and the strong adaptability of *H. hamabo* to stressed environments, genome-wide identification and analysis of the MYB transcription factor family in H. hamabo were performed for the first time in this study. The search for MYB family members in the *H. hamabo* genome led to the identification of 204 putative HhMYB family members. The HhMYB family was clustered into 28 subfamilies with AtMYBs according to phylogenetics and their motif patterns. In addition, we found that the 204 *HhMYB* genes were unevenly distributed across the 46 chromosomes of *H. hamabo*. Expression analysis with transcriptomic data and qRT-PCR showed that the candidate HhMYB family displays a wide range of sensitivities to abiotic stress and hormones. Several *HhMYB*s were involved in the plant responses to both saline and drought stresses to varying degrees, indicating that these *HhMYB*s may have regulatory functions in tolerance to saline and drought stress and deserve further investigation. Our results provide a foundation for further researching MYB gene functions in *H. hamabo.*

## Data Availability

No new data were created or analyzed in this study. Data sharing is not applicable to this article.

## Supplementary Materials

The following are available online at https://www.mdpi.com/article/10.3390/plants12071429/s1. Figure S1: Phylogeny (a), conserved motif (b), and gene structure (c) analyses of HhMYBs; Figure S2: Chromosome distribution of *HhMYB*s; Table S1: Primer sequences for qRT-PCR; Table S2: List of the MYB family genes in *H. hamabo.*

## References

[1] Sakhanokho H.F., Islam-Faridi N., Babiker E.M., Nelson C.D., Stringer S.J., Adamczyk J.J. (2020). Determination of nuclear DNA content, ploidy, and fish location of ribosomal DNA in hibiscus hamabo. Sci. Hortic..

[2] Wang Z., Xue J.-Y., Hu S.-Y., Zhang F., Yu R., Chen D., Van de Peer Y., Jiang J., Song A., Ni L. (2022). The genome of *Hibiscus hamabo* reveals its adaptation to saline and waterlogged habitat. Hortic. Res..

[3] Yan A.L., Qi Y.T., Li D.W. (2014). In Current status of Hangjiahu plain wetlands resources and proposals for protection and management. Adv. Mater. Res..

[4] Ni L., Wang Z., Liu X., Wu S., Hua J., Liu L., Yin Y., Li H., Gu C. (2022). Genome-wide study of the GRAS gene family in *Hibiscus hamabo* sieb. Et zucc and analysis of HhGRAS14-induced drought and salt stress tolerance in *Arabidopsis*. Plant Sci..

[5] Ni L., Wang Z., Liu L., Guo J., Li H., Gu C. (2019). Selection and verification of candidate reference genes for gene expression by quantitative RT-PCR in *Hibiscus hamabo* sieb. Et zucc. Trees.

[6] Wang Z., Ni L., Guo J., Liu L., Li H., Yin Y., Gu C. (2020). Phylogenetic and transcription analysis of *Hibiscus hamabo* sieb. Et zucc. WRKY transcription factors. DNA Cell Biol..

[7] Ni L., Wang Z., Fu Z., Liu D., Yin Y., Li H., Gu C. (2021). Genome-wide analysis of basic helix-loop-helix family genes and expression analysis in response to drought and salt stresses in *Hibiscus hamabo* sieb. Et zucc. Int. J. Mol. Sci..

[8] Ni L., Wang Z., Liu X., Wu S., Hua J., Yin Y., Li H., Gu C. (2021). Transcriptome analysis of salt stress in *Hibiscus hamabo* sieb. Et zucc based on pacbio full-length transcriptome sequencing. Int. J. Mol. Sci..

[9] Shi Q., Bao X.W., Hua J.F., Yu C.G., Yin Y.L., Lu Z.G. (2019). Effects of drought stress and recovery on photosynthesis and physiological characteristics of *Hibiscus hamabo*. J. Appl. Ecol..

[10] Du H., Zhang L., Liu L., Tang X.-F., Yang W.-J., Wu Y.-M., Huang Y.-B., Tang Y.-X. (2009). Biochemical and molecular characterization of plant MYB transcription factor family. Biochemistry.

[11] Li J., Han G., Sun C., Sui N. (2019). Research advances of MYB transcription factors in plant stress resistance and breeding. Plant Signal. Behav..

[12] Wang Y., Zhang Y., Fan C., Wei Y., Meng J., Li Z., Zhong C. (2021). Genome-wide analysis of MYB transcription factors and their responses to salt stress in *Casuarina equisetifolia*. BMC Plant Biol..

[13] Baldoni E., Genga A., Cominelli E. (2015). Plant MYB transcription factors: Their role in drought response mechanisms. Int. J. Mol. Sci..

[14] Smita S., Katiyar A., Chinnusamy V., Pandey D.M., Bansal K.C. (2015). Transcriptional regulatory network analysis of MYB transcription factor family genes in rice. Front. Plant Sci..

[15] Ganesan G., Sankararamasubramanian H., Harikrishnan M., Ashwin G., Parida A. (2012). A MYB transcription factor from the grey mangrove is induced by stress and confers NaCl tolerance in tobacco. J. Exp. Bot..

[16] Dubos C., Stracke R., Grotewold E., Weisshaar B., Martin C., Lepiniec L. (2010). MYB transcription factors in *Arabidopsis*. Trends Plant Sci..

[17] Arce-Rodríguez M.L., Martínez O., Ochoa-Alejo N. (2021). Genome-wide identification and analysis of the MYB transcription factor gene family in chili pepper (*Capsicum* spp.). Int. J. Mol. Sci..

[18] Wang J., Liu Y., Tang B., Dai X., Xie L., Liu F., Zou X. (2020). Genome-wide identification and capsaicinoid biosynthesis-related expression analysis of the R2R3-MYB gene family in *Capsicum annuum* L.. Front. Genet..

[19] Kumar S., Stecher G., Tamura K. (2016). MEGA7: Molecular evolutionary genetics analysis version 7.0 for bigger datasets. Mol. Biol. Evol..

[20] Hu B., Jin J., Guo A.-Y., Zhang H., Luo J., Gao G. (2015). GSDS 2.0: An upgraded gene feature visualization server. Bioinformatics.

[21] Krzywinski M., Schein J., Birol I., Connors J., Gascoyne R., Horsman D., Jones S.J., Marra M.A. (2009). Circos: An information aesthetic for comparative genomics. Genome Res..

[22] Wang Z., Ni L., Hua J., Liu L., Yin Y., Li H., Gu C. (2021). Transcriptome analysis reveals regulatory framework for salt and drought tolerance in *Hibiscus hamabo* siebold & zuccarini. Forests.

[23] Wang X., Niu Y., Zheng Y. (2021). Multiple functions of MYB transcription factors in abiotic stress responses. Int. J. Mol. Sci..

[24] Ambawat S., Sharma P., Yadav N.R., Yadav R.C. (2013). MYB transcription factor genes as regulators for plant responses: An overview. Physiol. Mol. Biol. Plants.

[25] Cao Y., Li K., Li Y., Zhao X., Wang L. (2020). MYB transcription factors as regulators of secondary metabolism in plants. Biology.

[26] Salih H., Gong W., He S., Sun G., Sun J., Du X. (2016). Genome-wide characterization and expression analysis of MYB transcription factors in *Gossypium hirsutum*. BMC Genet..

[27] Li J., Liu H., Yang C., Wang J., Yan G., Si P., Bai Q., Lu Z., Zhou W., Xu L. (2020). Genome-wide identification of MYB genes and expression analysis under different biotic and abiotic stresses in *Helianthus annuus* L.. Ind. Crops Prod..

[28] Katiyar A., Smita S., Lenka S.K., Rajwanshi R., Chinnusamy V., Bansal K.C. (2012). Genome-wide classification and expression analysis of MYB transcription factor families in rice and *Arabidopsis*. BMC Genom..

[29] Abbas F., Ke Y., Zhou Y., Yu Y., Waseem M., Ashraf U., Wang C., Wang X., Li X., Yue Y. (2021). Genome-wide analysis reveals the potential role of MYB transcription factors in floral scent formation in *Hedychium coronarium*. Front. Plant Sci..

[30] Zhang P., Wang R., Yang X., Ju Q., Li W., Lü S., Tran L.S.P., Xu J. (2020). The R2R3-MYB transcription factor *AtMYB49* modulates salt tolerance in *Arabidopsis* by modulating the cuticle formation and antioxidant defence. Plant Cell Environ..

[31] Park M.Y., Kang J.-y., Kim S.Y. (2011). Overexpression of *AtMYB52* confers ABA hypersensitivity and drought tolerance. Mol. Cells.

[32] Hussain S.S., Kayani M.A., Amjad M. (2011). Transcription factors as tools to engineer enhanced drought stress tolerance in plants. Biotechnol. Prog..

[33] Qin Y., Wang M., Tian Y., He W., Han L., Xia G. (2012). Over-expression of *TaMYB33* encoding a novel wheat MYB transcription factor increases salt and drought tolerance in *Arabidopsis*. Mol. Biol. Rep..

[34] Ma Q., Dai X., Xu Y., Guo J., Liu Y., Chen N., Xiao J., Zhang D., Xu Z., Zhang X. (2009). Enhanced tolerance to chilling stress in *OsMYB3R-2* transgenic rice is mediated by alteration in cell cycle and ectopic expression of stress genes. Plant Physiol..

[35] Abe H., Urao T., Ito T., Seki M., Shinozaki K., Yamaguchi-Shinozaki K. (2003). Arabidopsis *AtMYC2* (*bHLH*) and *AtTMYB2* (*MYB*) function as transcriptional activators in abscisic acid signaling. Plant Cell.

[36] Sun W., Ma Z., Chen H., Liu M. (2019). MYB gene family in potato (*Solanum tuberosum* L.): Genome-wide identification of hormone-responsive reveals their potential functions in growth and development. Int. J. Mol. Sci..

