# Supplementary material for: Genome-Wide Identification and Analysis of MYB Transcription Factor Family in Hibiscus hamabo
Source: Plants (Basel). 2023 Mar 23;12(7):1429. doi: 10.3390/plants12071429 (PMC10096737; doi:10.3390/plants12071429)
Supplement: Supplementary file 1 [file plants-12-01429-s001.zip › Table S1.pdf]

Table S1. Primer sequences for qRT-PCR.

Gene ID	Gene Name	Primer sequence
HhMYB5	*Hha032085*	F:CAGCCGCTTAAAAGATCGGC<br>R:AACCGGTCATTGCTATCGGG
HhMYB7	*Hha046916*	F:CGGCGTCTTCTTCTGACGAT<br>R:GTTCCTCGCCTGTGTTGACT
HhMYB46	*Hha076724*	F:TGCCCATGCAAAGCTATGGT<br>R:GTCGAACTCGAAGACGGTGT
HhMYB116	*Hha093713*	F:GCTTCTATTTGCGGAGTGGC<br>R:GGATAAATCCGTCGGAGGCA
HhMYB93	*Hha036320*	F:AATAGCGGCAACGTACGGAT<br>R:AACTGTTGCTGCTTTGTGGC
HhMYB75	*Hha038203*	F:AGCCGTGAACCCTTGTGAAA<br>R:CCTTGCTGCATGACTCCGTA
HhMYB157	*Hha040476*	F:GTCGTGGCTTCTTCAATCGC<br>R:CAGACCAGTACGGAGAAGCG
