# Supplementary material for: Genome-Wide Identification and Analysis of MYB Transcription Factor Family in Hibiscus hamabo
Source: Plants (Basel). 2023 Mar 23;12(7):1429. doi: 10.3390/plants12071429 (PMC10096737; doi:10.3390/plants12071429)
Supplement: Supplementary file 1 [file plants-12-01429-s001.zip › Table S2.pdf]

Table S2. List of the MYB family genes in *H. hamabo*.

Gene name	Gene ID	Protein length/aa	Moleculer mass/ku	PI	Gene name	Gene ID	Protein length/aa	Moleculer mass/ku	PI
**Hha001228**	*HhMYB1*	325	35.92	9.13	**Hha046546**	*HhMYB103*	430	47.95	7.52
**Hha043554**	*HhMYB2*	313	34.31	10.06	**Hha055887**	*HhMYB104*	407	45.31	7.02
**Hha091305**	*HhMYB3*	302	32.86	9.04	**Hha012986**	*HhMYB105*	311	34.60	5.44
**Hha084654**	*HhMYB4*	300	32.81	8.05	**Hha096592**	*HhMYB106*	311	34.15	5.19
**Hha032085**	*HhMYB5*	313	34.38	8.74	**Hha082377**	*HhMYB107*	300	33.16	6.37
**Hha047495**	*HhMYB6*	308	33.49	6.52	**Hha028223**	*HhMYB108*	311	34.36	5.06
**Hha046916**	*HhMYB7*	314	34.57	5.76	**Hha063318**	*HhMYB109*	326	36.25	5.06
**Hha052759**	*HhMYB8*	311	33.78	6.67	**Hha080482**	*HhMYB110*	305	33.67	6.3
**Hha072229**	*HhMYB9*	419	45.42	5.91	**Hha077477**	*HhMYB111*	304	33.80	6.37
**Hha075298**	*HhMYB10*	416	45.29	6.51	**Hha031648**	*HhMYB112*	319	35.45	5.98
**Hha003980**	*HhMYB11*	406	44.40	5.84	**Hha093430**	*HhMYB113*	309	34.58	6.12
**Hha025178**	*HhMYB12*	402	43.98	6.33	**Hha093431**	*HhMYB114*	313	35.21	6.28
**Hha090844**	*HhMYB13*	399	43.16	6.07	**Hha061965**	*HhMYB115*	331	37.10	6.79
**Hha008339**	*HhMYB14*	308	33.60	8.81	**Hha093713**	*HhMYB116*	302	34.29	6.9
**Hha016640**	*HhMYB15*	318	35.05	8.8	**Hha013266**	*HhMYB117*	336	37.65	5.6
**Hha031294**	*HhMYB16*	348	38.88	7.82	**Hha086362**	*HhMYB118*	345	38.17	6.19
**Hha045589**	*HhMYB17*	335	37.40	8.08	**Hha030519**	*HhMYB119*	355	39.52	4.93
**Hha087220**	*HhMYB18*	377	42.01	7.22	**Hha077811**	*HhMYB120*	343	38.16	5.18
**Hha078618**	*HhMYB19*	366	40.89	6.74	**Hha057760**	*HhMYB121*	320	36.35	7.8
**Hha048791**	*HhMYB20*	399	44.06	7.13	**Hha088058**	*HhMYB122*	318	35.47	5.7
**Hha014018**	*HhMYB21*	412	45.75	7.84	**Hha020480**	*HhMYB123*	300	34.06	5.24
**Hha016966**	*HhMYB22*	343	37.50	8.82	**Hha094789**	*HhMYB124*	300	34.14	4.78
**Hha068516**	*HhMYB23*	310	34.17	7.94	**Hha013750**	*HhMYB125*	307	35.30	6.72
**Hha054120**	*HhMYB24*	357	38.91	8.73	**Hha070672**	*HhMYB126*	305	34.90	6.78
**Hha034145**	*HhMYB25*	316	34.79	9.71	**Hha102430**	*HhMYB127*	302	34.20	5.97
**Hha069012**	*HhMYB26*	332	36.38	9.1	**Hha103223**	*HhMYB128*	354	40.16	6.86
**Hha034644**	*HhMYB27*	339	37.31	9.06	**Hha062347**	*HhMYB129*	314	34.81	8.49
**Hha070962**	*HhMYB28*	342	37.57	8.11	**Hha014006**	*HhMYB130*	317	35.61	8.96
**Hha022357**	*HhMYB29*	342	38.31	9.38	**Hha070950**	*HhMYB131*	311	35.14	7.68
**Hha025478**	*HhMYB30*	327	37.08	10.01	**Hha032978**	*HhMYB132*	319	35.80	7.95
**Hha050768**	*HhMYB31*	324	36.42	9.81	**Hha032979**	*HhMYB133*	321	36.25	7.63
**Hha040614**	*HhMYB32*	315	35.42	9.42	**Hha070951**	*HhMYB134*	345	38.73	6.57
**Hha043344**	*HhMYB33*	342	38.33	9.5	**Hha014007**	*HhMYB135*	353	39.79	8.23
**Hha041580**	*HhMYB34*	469	52.21	9.04	**Hha072615**	*HhMYB136*	341	38.07	6.84
**Hha044344**	*HhMYB35*	467	52.18	8.56	**Hha074943**	*HhMYB137*	397	45.46	6.16
**Hha088807**	*HhMYB36*	415	47.43	6.72	**Hha093132**	*HhMYB138*	342	38.36	5.95
**Hha064189**	*HhMYB37*	501	56.07	7.25	**Hha094646**	*HhMYB139*	332	37.34	6.12
**Hha101247**	*HhMYB38*	439	49.22	6.51	**Hha053135**	*HhMYB140*	311	34.81	4.51
**Hha000993**	*HhMYB39*	412	44.68	6.37	**Hha015768**	*HhMYB141*	308	33.83	5.92
**Hha073622**	*HhMYB40*	337	38.45	6.14	**Hha072711**	*HhMYB142*	327	36.48	4.83
**Hha043811**	*HhMYB41*	365	41.45	7.41	**Hha025619**	*HhMYB143*	338	37.41	4.81
**Hha101571**	*HhMYB42*	319	36.16	5.19	**Hha057104**	*HhMYB144*	356	39.41	4.59
**Hha022014**	*HhMYB43*	307	35.05	9.18	**Hha052068**	*HhMYB145*	394	43.92	4.76
**Hha041108**	*HhMYB44*	360	41.02	6.31	**Hha001515**	*HhMYB146*	312	35.23	6.17
**Hha038327**	*HhMYB45*	358	40.62	7.03	**Hha070099**	*HhMYB147*	307	34.92	7.15

**Hha076724**	*HhMYB46*	357	39.60	8.54	**Hha010621**	*HhMYB148*	321	35.72	6.8
**Hha097018**	*HhMYB47*	344	38.23	8.46	**Hha064504**	*HhMYB149*	316	35.25	7.02
**Hha026221**	*HhMYB48*	305	33.96	9.45	**Hha058537**	*HhMYB150*	321	35.51	6.44
**Hha020980**	*HhMYB49*	309	34.28	8.97	**Hha012814**	*HhMYB151*	325	36.10	6.51
**Hha024023**	*HhMYB50*	334	36.84	6.76	**Hha079347**	*HhMYB152*	332	37.35	6.84
**Hha077962**	*HhMYB51*	317	35.45	6.61	**Hha083550**	*HhMYB153*	322	35.87	8.66
**Hha092174**	*HhMYB52*	336	37.47	7.79	**Hha083553**	*HhMYB154*	320	35.58	7.73
**Hha089006**	*HhMYB53*	344	38.45	6.62	**Hha026956**	*HhMYB155*	323	36.40	5.24
**Hha036803**	*HhMYB54*	324	35.95	7.39	**Hha061862**	*HhMYB156*	333	37.20	6.24
**Hha022861**	*HhMYB55*	339	37.73	8.16	**Hha040476**	*HhMYB157*	327	35.91	6.79
**Hha000608**	*HhMYB56*	330	36.08	8.04	**Hha050913**	*HhMYB158*	303	33.36	6.99
**Hha041463**	*HhMYB57*	322	36.31	7.05	**Hha038106**	*HhMYB159*	321	35.70	7.23
**Hha012903**	*HhMYB58*	330	37.02	7.83	**Hha040715**	*HhMYB160*	312	34.62	7.41
**Hha077517**	*HhMYB59*	308	35.01	8.19	**Hha053527**	*HhMYB161*	349	39.03	7.28
**Hha086281**	*HhMYB60*	314	35.53	7.96	**Hha100252**	*HhMYB162*	328	36.67	6.89
**Hha090945**	*HhMYB61*	324	36.04	6.08	**Hha016644**	*HhMYB163*	350	38.93	6.08
**Hha084545**	*HhMYB62*	318	35.24	6.39	**Hha017002**	*HhMYB164*	340	38.06	6.96
**Hha060051**	*HhMYB63*	323	35.94	7.17	**Hha054054**	*HhMYB165*	356	40.02	6.32
**Hha006683**	*HhMYB64*	352	39.17	7.43	**Hha006814**	*HhMYB166*	304	34.16	6.52
**Hha066713**	*HhMYB65*	352	39.29	5.42	**Hha066553**	*HhMYB167*	303	33.87	7.81
**Hha042445**	*HhMYB66*	305	34.11	6.36	**Hha035591**	*HhMYB168*	307	34.39	6.53
**Hha035773**	*HhMYB67*	367	40.69	7.77	**Hha014085**	*HhMYB169*	326	36.57	6.68
**Hha063988**	*HhMYB68*	354	39.37	6.25	**Hha047390**	*HhMYB170*	319	35.11	7.84
**Hha057968**	*HhMYB69*	344	38.17	5.21	**Hha028064**	*HhMYB171*	314	34.60	7.47
**Hha039314**	*HhMYB70*	371	41.29	6.78	**Hha063023**	*HhMYB172*	303	33.43	7.44
**Hha043513**	*HhMYB71*	353	39.53	7.5	**Hha047011**	*HhMYB173*	308	34.37	8.79
**Hha042085**	*HhMYB72*	363	40.34	5.69	**Hha068060**	*HhMYB174*	329	36.47	7.12
**Hha010091**	*HhMYB73*	352	39.34	5.41	**Hha034650**	*HhMYB175*	300	33.62	7.39
**Hha040810**	*HhMYB74*	338	37.89	7.99	**Hha068502**	*HhMYB176*	304	34.32	9.17
**Hha038203**	*HhMYB75*	368	41.60	6.67	**Hha093998**	*HhMYB177*	328	36.63	7.18
**Hha050568**	*HhMYB76*	357	40.25	8.22	**Hha105322**	*HhMYB178*	306	33.96	7.9
**Hha073035**	*HhMYB77*	346	37.81	7.91	**Hha058761**	*HhMYB179*	305	34.30	7.79
**Hha074485**	*HhMYB78*	350	38.27	8.38	**Hha092431**	*HhMYB180*	329	36.98	7.55
**Hha035047**	*HhMYB79*	355	39.23	6.25	**Hha035041**	*HhMYB181*	327	36.40	8.01
**Hha102191**	*HhMYB80*	323	35.39	8.03	**Hha009089**	*HhMYB182*	548	59.75	6.51
**Hha004331**	*HhMYB81*	343	37.57	7.09	**Hha082719**	*HhMYB183*	539	59.19	6.58
**Hha014005**	*HhMYB82*	348	38.24	7.91	**Hha080924**	*HhMYB184*	547	59.77	6.8
**Hha032977**	*HhMYB83*	357	39.72	6.99	**Hha096220**	*HhMYB185*	540	59.29	6.43
**Hha054713**	*HhMYB84*	305	33.93	8.68	**Hha099691**	*HhMYB186*	1731	196.98	8.95
**Hha031270**	*HhMYB85*	326	36.80	8.76	**Hha018036**	*HhMYB187*	511	56.62	5.06
**Hha000806**	*HhMYB86*	337	37.14	7.78	**Hha033555**	*HhMYB188*	483	53.21	4.78
**Hha028461**	*HhMYB87*	342	37.69	7.01	**Hha085174**	*HhMYB189*	477	52.84	4.67
**Hha073372**	*HhMYB88*	392	43.43	7.04	**Hha015545**	*HhMYB190*	555	60.49	4.92
**Hha103789**	*HhMYB89*	364	40.14	6.76	**Hha065583**	*HhMYB191*	504	55.35	4.93
**Hha087796**	*HhMYB90*	353	39.35	7.35	**Hha087597**	*HhMYB192*	550	60.81	4.74
**Hha020187**	*HhMYB91*	300	33.27	7.66	**Hha065213**	*HhMYB193*	536	58.15	4.66
**Hha001626**	*HhMYB92*	321	36.02	5.41	**Hha037790**	*HhMYB194*	632	69.51	5.04
**Hha036320**	*HhMYB93*	380	42.82	7.8	**Hha102505**	*HhMYB195*	555	60.18	4.51
**Hha063483**	*HhMYB94*	371	41.48	8.39	**Hha019843**	*HhMYB196*	764	83.81	4.75

**Hha014976**	*HhMYB95*	385	42.87	5.71	**Hha019607**	*HhMYB197*	372	41.00	6.78
**Hha062098**	*HhMYB96*	409	45.82	7.13	**Hha059414**	*HhMYB198*	307	34.16	5.95
**Hha088297**	*HhMYB97*	397	44.35	6.77	**Hha016482**	*HhMYB199*	388	42.59	6.71
**Hha027114**	*HhMYB98*	418	46.73	7.02	**Hha049662**	*HhMYB200*	1158	128.85	5.25
**Hha023472**	*HhMYB99*	409	45.74	6.58	**Hha061368**	*HhMYB201*	1011	111.44	5.07
**Hha079141**	*HhMYB100*	401	44.95	6.73	**Hha059990**	*HhMYB202*	509	56.18	9.03
**Hha095515**	*HhMYB101*	425	47.89	6.82	**Hha056109**	*HhMYB203*	555	61.26	7.92
**Hha047854**	*HhMYB102*	442	49.04	8.47	**Hha057292**	*HhMYB204*	451	50.45	9.01
