# Supplementary figures and images for: Genome-Wide Identification and Analysis of MYB Transcription Factor Family in Hibiscus hamabo
Source: Plants (Basel). 2023 Mar 23;12(7):1429. doi: 10.3390/plants12071429 (PMC10096737; doi:10.3390/plants12071429)

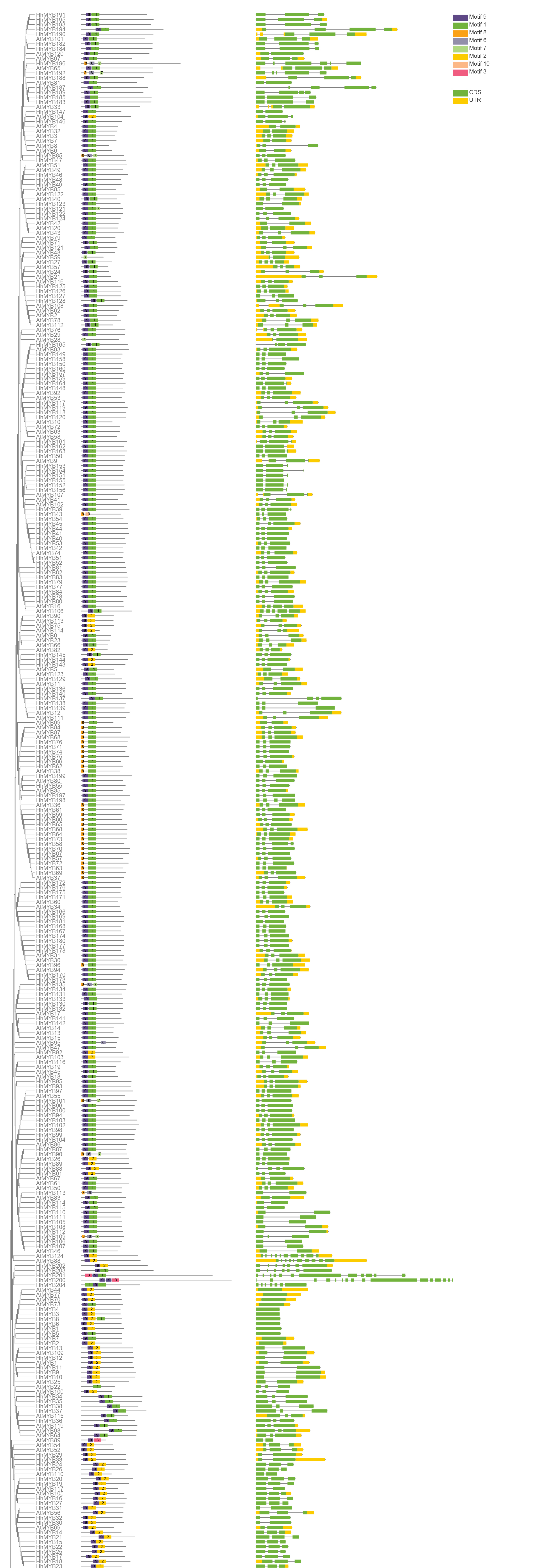

Supplement: Supplementary file 1 [file plants-12-01429-s001.zip › Figure S1.jpg]

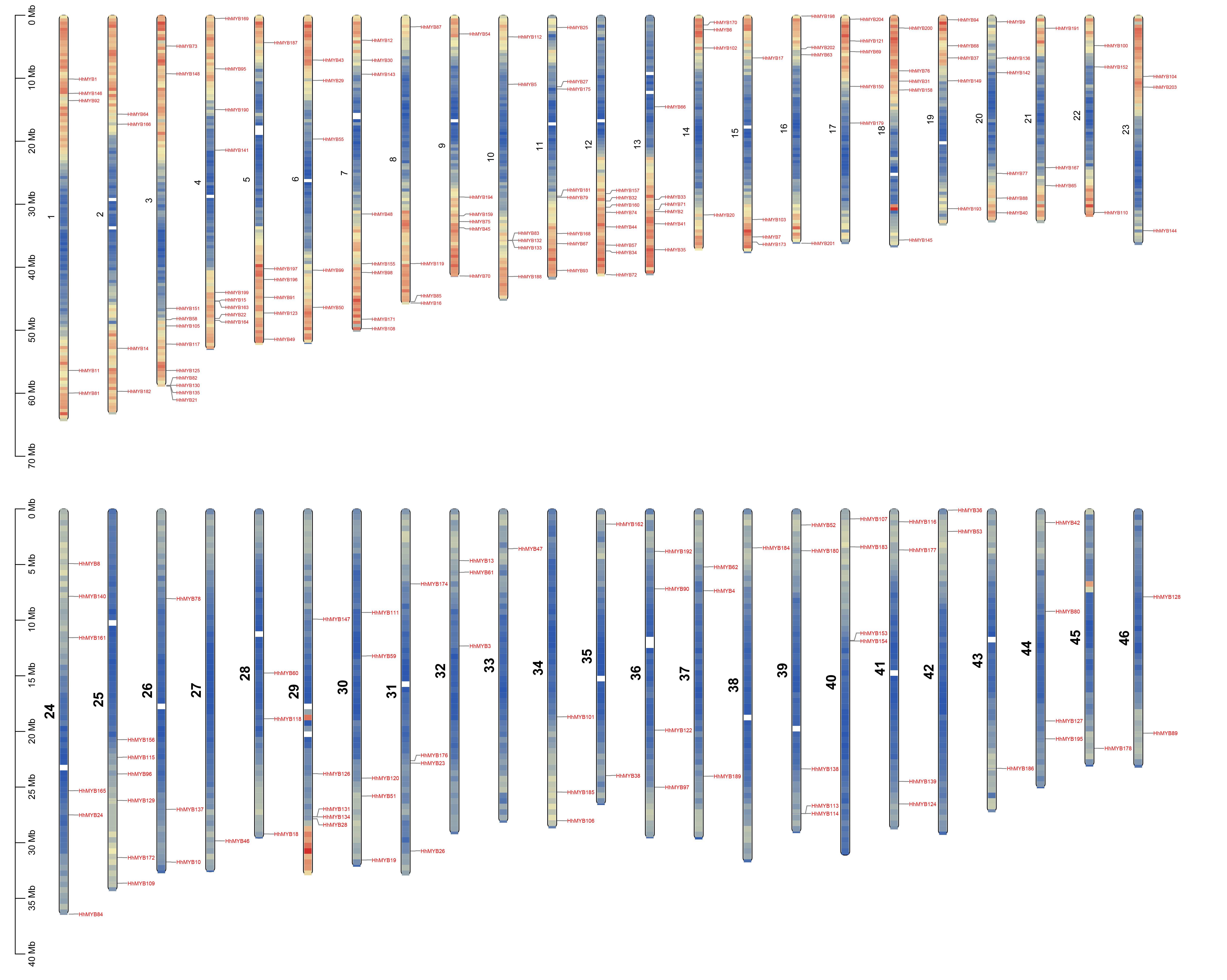

Supplement: Supplementary file 1 [file plants-12-01429-s001.zip › Figure S2.jpg]
